# Zinc-Containing Over-The-Counter Product Causing Sideroblastic Anemia and Neutropenia

**DOI:** 10.7759/cureus.59796

**Published:** 2024-05-07

**Authors:** Marshall Patrick Stagg, Jennifer Miatech, Baseera Majid, Ravali Polala

**Affiliations:** 1 Hematology and Oncology, Our Lady of the Lake Regional Hospital, Baton Rouge, USA; 2 Hematology and Oncology, Our Lady of the Lake Cancer Institute, Baton Rouge, USA; 3 Hematology and Oncology, Our Lady of the Lake Regional Medical Center, Baton Rouge, USA; 4 Hematology and Oncology, Louisiana State University School of Medicine, New Orleans, USA

**Keywords:** zinc-induced hypocupremia, zinc, sideroblastic anemia, neutropenia, copper

## Abstract

Sideroblastic anemia is characterized by anemia, granulocytopenia, and bone marrow findings of vacuolated precursors and ringed sideroblasts. Zinc-induced copper deficiency can present as sideroblastic anemia and neutropenia. We report the case of a previously healthy 74-year-old female who presented with newly discovered sideroblastic anemia as a result of an over-the-counter oral vitamin and mineral supplement. Serum analysis revealed increased zinc levels, decreased copper levels, and a decrease in ceruloplasmin. Bone marrow evaluation revealed ringed sideroblasts and cytoplasmic vacuolization in myeloid precursors. She demonstrated improvement in her hematologic profile with discontinuation of the over-the-counter product and administration of oral copper supplementation. This case highlights the importance of sideroblastic anemia recognition and careful medication review, including over-the-counter supplements.

## Introduction

Sideroblastic anemia encompasses a group of heterogeneous congenital and acquired disorders characterized by anemia and the presence of ring sideroblasts in the bone marrow. Acquired sideroblastic anemia is most commonly associated with myelodysplastic disorders (MDS), toxins/drugs, and, rarely, nutritional deficiencies [1]. Copper serves as an essential cofactor for several essential human enzymes such as superoxide dismutase, cytochrome‐c oxidase, and ceruloplasmin [1]. Copper deficiency or hypocupremia is known to cause sideroblastic anemia, neutropenia, and neurological complications.

Hypocupremia can occur in the setting of decreased oral intake, impaired absorption, or excessive gastrointestinal or urinary losses. Prolonged and excessive exposure to zinc is known to impair the absorption of copper from the gastrointestinal tract. Metallothionein is an intracellular ligand of mucosal cells that plays a crucial role in the absorption of both copper and zinc. Excess levels of zinc consumption stimulate metallothionein synthesis in enterocytes with a stronger affinity for copper, thus resulting in decreased zinc absorption.

Zinc-induced copper deficiency is a rare and difficult-to-recognize condition. Cases have previously been attributed to the use of oral, topical, and enteral products, including over-the-counter supplements and denture creams [1-3]. Earlier recognition of hypocupremia helps avoid the potential development of neurological deficits and persistent cytopenias. We present a case of a 74-year-old Caucasian female who presented with sideroblastic anemia and neutropenia caused by zinc-induced copper deficiency through the use of over-the-counter vitamin and mineral supplement.

## Case presentation

A 74-year-old Caucasian female initially presented to her primary care physician’s office with complaints of progressive fatigue, generalized weakness, and shortness of breath for two weeks. The patient was instructed to present to the emergency department after laboratory studies revealed a hemoglobin of 6.1 g/dL (normal range: 11.2-15.7 g/dL). She denied any melena, hematochezia, hematemesis, hemoptysis, or any other signs of bleeding. She also denied a history of anemia or previous blood transfusions. The patient has a past medical history of hypertension, type 2 diabetes mellitus, hyperlipidemia, stage IV chronic kidney disease with a baseline creatinine of 1.7 g/dL, and a history of invasive breast cancer that was successfully treated with lumpectomy and radiation with hormonal therapy 14 years prior. She had never received systemic chemotherapy. 

Upon presentation to the emergency department, the patient had a blood pressure of 154/85 mmHg, pulse of 95 beats per minute, temperature of 97.7°Celsius, a respiratory rate of 17 per minute, and oxygen saturation 98% on room air. On physical examination, she was pale in appearance and with no other significant findings. Laboratory studies entailing a complete blood picture revealed a white blood count of 1.94 x 10^3^/µL with an absolute lymphocyte count of 710/μL and absolute neutrophil count of  900/μL, hemoglobin of 5.7 g/dL with a mean corpuscular volume of 101 fL, a low corrected reticulocyte count of 0.08%, and a platelet count of 231 x 10^3^/µL. The peripheral blood smear showed moderate normochromic, normocytic anemia (Table 1). She was also found to have an elevated creatinine of 2.13 mg/dL with no other abnormalities on her complete metabolic profile. Serum folate level was 11.2 ng/mL (normal range: 3.1-17.5 ng/mL) and her vitamin B12 level at 324 pg/mL (normal range: 193-986 pg/mL). Iron studies revealed a serum ferritin level of 305.5 ng/mL (normal range: 8.0-252 ng/mL), serum iron of 84 µg/dL (normal range: 50-170 µg/dL), total iron binding capacity of 268 µg/dL (normal range: 250-480 µg/dL), and an iron saturation of 31.3% (normal range: 20-50%).

**Table 1:** Interval laboratory studies of a 74-year-old female over the course of two months with noticeable improvement after discontinuing the zinc-containing over-the-counter supplement

Laboratory studies	May 4, 2021	June 15, 2021	July 20, 2021	Reference ranges
Hemoglobin	5.7	6.5	11.4	11.2-15.7 g/dL
White blood count	1.94	2.46	8.14	4-11 K/μL
Absolute neutrophil count	900	1320	5960	1,600–7,420 K/μL
Serum copper	-	6	67	80-158 μg/dL
Serum ceruloplasmin	-	< 3.0	14.4	19-39 mg/dL
Zinc	-	188	151	44-115 μg/dL

The patient was transfused with 2 units of packed red blood cells with an improvement in her hemoglobin to 7.5 g/dL. The patient’s acute kidney injury was attributed to dehydration and improved to her baseline creatinine with intravenous fluids. She was scheduled for follow-up with hematology in one week for a bone marrow biopsy and was discharged home.

**Figure 1:**
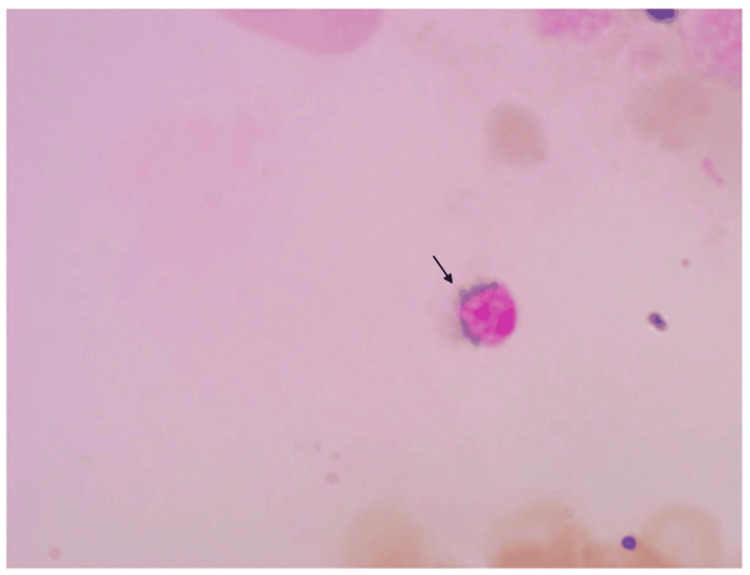
Bone marrow biopsy sample of a 74-year-old female showing a ringed sideroblast on Perls Prussian blue stain (H&E, 50x), i.e., blue color staining the erythroid precursor. H&E, hematoxylin and eosin

**Figure 2:**
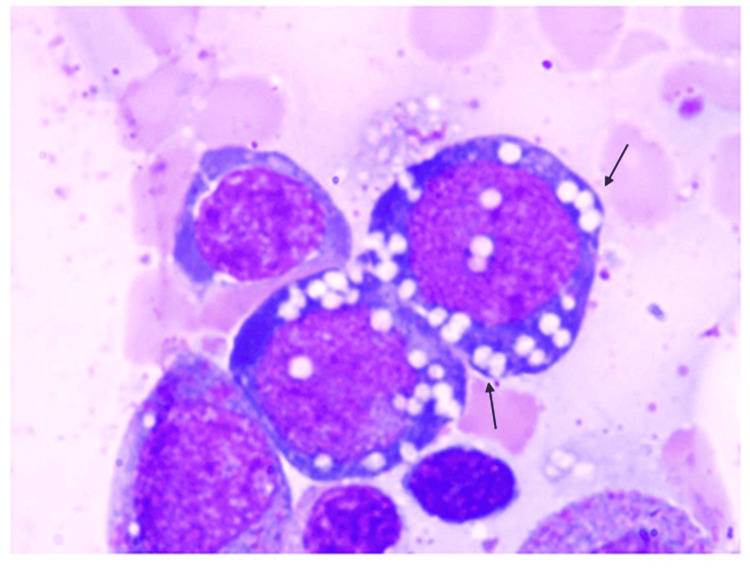
Bone marrow biopsy sample of a 74-year-old female demonstrating cytoplasmic vacuolization of myeloid precursors (H&E, 100x). H&E, hematoxylin and eosin

The bone marrow biopsy performed the following week revealed a hypercellular bone marrow with myeloid and erythroid hyperplasia, left shifted myelopoiesis, and erythropoiesis. Minimal changes were seen in the erythroid series, with less than 10% dyserythropoiesis. No significant blast cells, dysplasia, atypical lymphoid, or plasma cell populations were identified. However, ringed sideroblasts were found to be high at approximately 15%, and cytoplasmic vacuolization in myeloid precursors was noted (Figures 1, 2). Flow cytometry and cytogenetic studies also demonstrated normal results. The red blood cells showed macrocytic anemia and neutropenia, leading to a diagnosis of sideroblastic anemia with no significant myelodysplasia. Further investigation for sideroblastic anemia with copper and ceruloplasmin serum levels was ordered. She was found to be persistently anemic during her outpatient visits with a repeat hemoglobin of 6.5 g/dL. Given the patient’s low reticulocyte count and severe, symptomatic anemia, 1 dose of erythropoietin and an additional 2 units of packed red blood cells were given as an outpatient while awaiting laboratory results.

The patient was found to have a decreased ceruloplasmin level of <3 mg/dL (normal range: 19.0-39.0 mg/dL) and copper level of  6 սg/dL (normal range: 80-158 սg/dL) with an elevated zinc level of 188 սg/dL (normal range: 80-158 սg/dL) on June 15, 2021. Further investigation into the patient’s current medications revealed that she had been taking the over-the-counter oral medication PreserVision twice daily, which contained 40 mg of zinc and 1 mg of copper. This medication was recommended by her ophthalmologist for the prevention of macular degeneration. The patient was instructed to discontinue the ocular vitamin and mineral supplement, and oral copper supplementation was initiated at 2 mg daily. After approximately four weeks, her copper, ceruloplasmin, and zinc levels began to normalize with an improvement of her hemoglobin to 11.4 g/dL and absolute neutrophil count to 5,960 K/μL. The patient has remained off of the over-the-counter product and is no longer requiring oral copper supplementation; she had a stable hemoglobin of 12.7 g/dL at the six-month follow-up.

## Discussion

Sideroblastic anemia comprises a wide spectrum of heritable and acquired erythropoietic disorders that result from abnormalities in heme synthesis and mitochondrial function. Sideroblastic anemia is characterized by anemia and the presence of ring sideroblasts and erythroblasts with iron-loaded mitochondria visualized by Prussian blue staining in the bone marrow. More commonly, it occurs in the setting of MDS or from exposure to toxins or drugs. Rarely, sideroblastic anemia occurs in the setting of copper deficiency. Hypocupremia can present with several hematological manifestations including sideroblastic anemia, leukopenia, and neutropenia, in addition to long-term neurological conditions. Sideroblastic anemia and neutropenia are the main hematologic features of hypocupremia. Several bone marrow findings have been attributed to copper deficiency including hypocellularity, erythroid hyperplasia, granulocytic hypoplasia, vacuolization of erythroid and myeloid precursors, and ringed sideroblasts [1].  Several mechanisms can contribute to the development of anemia and neutropenia due to hypocupremia, such as reduced absorption of iron from the intestines, decreased release of iron from the reticuloendothelial system, ineffective erythropoiesis and myelopoiesis due to diminished activity of copper‐containing enzymes, and increased destruction of erythrocytes [3,4]. 

Dietary copper intake is approximately 1 to 2 mg per day [5]. Copper is absorbed primarily in the stomach and proximal small intestine. Once absorbed, it becomes bound to plasma albumin and amino acids and is transported to the liver to become incorporated into the copper-containing protein ceruloplasmin, which serves to transport copper from the liver to peripheral tissues. Ceruloplasmin also serves a role in iron metabolism by facilitating the conversion of iron to allow binding by plasma transferrin. Excess copper is predominantly excreted into the bile. Hypocupremia can occur in the setting of decreased oral intake, impaired absorption, or excessive gastrointestinal and urinary losses. Prolonged and excessive exposure to zinc is known to impair the absorption of copper from the gastrointestinal tract [6]. 

Zinc is primarily absorbed within the duodenum and jejunum of the small intestine. The recommended dietary intake for zinc is approximately 8 mg/day for adult females and 11 mg/day for adult males. The risk of zinc-induced copper metabolism disturbance begins to occur when zinc ingestions exceed 50 mg/day, although it typically arises when greater than 150 mg/day is consumed [7]. The presence of excess zinc leads to the overproduction of metallothionein, a copper and zinc-binding ligand that is present in enterocytes. Due to increased metallothionein concentration in enterocytes, copper preferentially binds with the metallothionein. Except for the excessive intake of particular types of seafood, zinc excess is unlikely to be obtained primarily through diet alone. This leads to decreased absorption of copper into the systemic circulation. As the copper remains bound inside the enterocytes and is sloughed off, the stored metallothionein‐copper complex becomes excreted [8].  On the contrary, increasing oral copper intake would be unproductive in restoring the zinc-copper balance in the setting of excess zinc as the induced metallothionein continues to intercept the copper and reduce its absorption [9].

Zinc-induced hypocupremia is often difficult to diagnose and identify potential sources.  Several cases have been published in the literature attributing zinc excess to denture creams and occasionally over-the-counter supplements [1-3]. Diagnosis can be aided by measurement of a low ceruloplasmin and copper level in addition to an elevated zinc level, as was seen in our patient. Our patient had been taking two tablets daily of the over-the-counter supplement PreserVision, which contained 40 mg of zinc and 1 mg of copper per tablet. This supplement was recommended by an ophthalmologist for the prevention of macular degeneration. Zinc-containing supplements are often recommended by ophthalmologists as literature has suggested that antioxidant vitamin and mineral supplements may prevent cellular damage in the retina by reacting with free radicals that are produced in the process of light absorption [10]. No other sources of potential zinc excess could be identified from her diet or remaining medications, and her hematological profile improved after discontinuation of the supplement. 

## Conclusions

Hypocupremia due to zinc products can lead to sideroblastic anemia and neutropenia, imitating other severe hematological disorders. Timely consideration of potential copper deficiency and a thorough clinical history can help evade unnecessary interventions and prevent potentially irreversible neurological complications. Therefore, it is valuable to remember hypocupremia as a possible etiology in patients presenting with cytopenias. Prompt detection of hypocupremia avoids future morbidity of neurological deficits and a decline in quality of life due to cytopenias and deflects continued ineffective interventions. Over-the-counter supplements are prevalent and often not on patients' medication lists. Zinc-induced hypocupremia-related hematological complications are easily treatable with copper supplementation and cessation of the source of zinc excess.
